# A population-based survey on physical inactivity and leisure time physical activity among adults in Chiang Mai, Thailand, 2014

**DOI:** 10.1186/s13690-017-0210-z

**Published:** 2017-10-02

**Authors:** Sanhapan Thanamee, Kanokporn Pinyopornpanish, Apichai Wattanapisit, Suparerk Suerungruang, Kanittha Thaikla, Wichuda Jiraporncharoen, Chaisiri Angkurawaranon

**Affiliations:** 1 Department of Family Medicine, Faculty of Medicine, Chiang Mai University, Chiang Mai, 50200 Thailand; 2 School of Medicine, Walailak University, Nakhon Si Thammarat, 80161 Thailand; 3 Health Promotion Center Region 1, Chiang Mai, 50100 Thailand; 4 Research Institute for Health Sciences, Chiang Mai University, Chiang Mai, 50200 Thailand

**Keywords:** Public health, Physical activity, Leisure time physical activity, Epidemiology, Thailand

## Abstract

**Background:**

Reducing physical inactivity among the population is a challenge for many nations. Targeting leisure time physical activity (LTPA) may be useful in increasing overall physical activity as it is assumed it is associated with a higher degree of free choice and personal preference than physical activity at work and during travel. The study explored the prevalence of physical inactivity and focused on the overall level of energy expenditure and energy level spent during leisure time among those who were physically inactive and assessed the stages of change for LTPA among those who were physically inactive.

**Methods:**

A population-based survey was conducted in 2014 in Chiang Mai, Thailand using a stratified two-stage cluster sampling technique. The Global Physical Activity Questionnaire (GPAQ) was used to collect the data on physical activity. Sufficient levels of physical activity (PA) were defined as ≥150 min/week of moderate-intensity PA or ≥75 min/week of vigorous-intensity PA or ≥600 metabolic equivalent of task (MET)-minutes/week. Weighted analyses were used to estimate the prevalence of physical inactivity, the total energy expenditure and expenditure during LTPA as well as stages of change among the physically inactive population.

**Results:**

A total of 1744 people (808 men and 936 women), aged 15 to 64 years, participated in the study. We estimated that a quarter (26%) of the population were physically inactive. Physical inactivity was more commonly found among women than men in most age groups. LTPA contributed a small proportion of overall PA. On average, physically inactive men spent 132.8 MET-minutes/week and inactive women spent 208.2 MET-minutes/week in overall PA which is well below the 600 MET-minutes/week recommend by the World Health Organization. Around 75% of physically inactive people had no intention of engaging in regular LTPA.

**Conclusion:**

About a quarter of the investigative population were physically inactive. Most physically inactive members of the population participate in low levels of LTPA, but the majority has no intention of increasing PA during leisure time. A large-scale health promotion program is needed, and it should focus on an approach for the pre-contemplated population.

## Background

Physical inactivity or insufficient physical activity (PA) is defined as less than 150 min/week of moderate-intensity PA accumulated across work, home, transport and leisure time activities [1]. It contributes to a global increase in health risks. Worldwide, about 23% of people aged 18 and above were physically inactive in 2010 (men 20% and women 27%) [2] . The World Health Organization (WHO) states that physical inactivity causes 3.2 million deaths each year [3]. Physical inactivity is a risk factor for many major non-communicable diseases (NCDs), for instance, coronary heart disease; type 2 diabetes mellitus; breast cancer; and colon cancer and is associated with increasing all-cause mortality [4]. In contrast, sufficient PA can reduce mortalities including from cardiovascular mortality, cancer mortality and all-cause mortality [5, 6]. Consequently, in 2010, the WHO provided the Global Recommendations on Physical Activity for Health to promote PA among children, adults, and older adults [7].

In Thailand, approximately 28.4% of adults are physically inactive [8]. Thai people spend 2 h a day involved in PA and about 13 h in sedentary behaviours [9]. About one-fifth (17.1%) of Thai adults were overweight [10] while prevalence of class I obesity and class II obesity were 26.0% and 9.0%, respectively [11].

Reducing physical inactivity is a challenge for many nations. According to the WHO guidelines, PA is classified into 3 domains: work; travel and recreation, the latter can be referred to as leisure time physical activity (LTPA). Given the same intensity, duration and frequency of an activity, these 3 types of PA are comparable in terms of energy expenditure [12]. However, targeting LTPA is an attractive approach to increase the overall PA and to decrease the prevalence of physical inactivity in the population as it is assumed to be associated with a higher degree of free choice and personal preference than PA at work and during travel [13]. In other words, interventions to encourage LTPA might be easier to promote and implement than interventions to promote PA at work and during travel as PA at work and during travel may depend on less modifiable factors such as distance to the workplace and characteristics of work.

To increase LTPA, a better understanding of the epidemiology and characteristics of physical inactivity and the contribution of LTPA to the overall PA level may be an important aspect for its promotion. In addition, previous studies show that an understanding of the stages of change to PA is also a key issue to consider [14, 15]. Not only does intention to change help predict the behaviour, studies have suggested that those in more advance stages of change for PA were also more likely to have higher levels of self-management and self-efficacy [15, 16]. In addition, understanding the stages of change (intention to change) may influence the efficacy of a health promotion programme, as those in different stages may require different forms of interventions [16, 17].

This study is a part of a larger survey on health risk behaviours carried out in 2014. A population-based survey was conducted to investigate the level of PA, especially LTPA, among people living in Chiang Mai, the most populated province in Northern Thailand. We aimed to explore the prevalence of physical inactivity and the contribution of LTPA to the overall level of PA across different age groups. We also focused on the overall level of energy expenditure as well as energy level spent during leisure time among those who were physically inactive and assessed the stages of change for LTPA among those who were physically inactive. The findings of the study will reflect the overall PA of the population. They will help to plan and monitor the need for and effectiveness of future interventions.

## Methods

### Study design

A stratified two-stage cluster sampling survey was conducted to investigate PA among people aged 15 to 64 years in Chiang Mai province in 2014. People who had lived in Chiang Mai for less than 3 months were excluded. The trained field researchers collected all data using face-to-face interviews and questionnaires. The Global Physical Activity Questionnaire (GPAQ) version 2 [12] in Thai language was used to assessed the level of physical activity. A study, investigated the reliability and validity of the GPAQ from nine countries, showed the moderate to substantial strength of its reliability (0.67 to 0.73); moderate to strong concurrent validity compared with the International Physical Activity Questionnaire (IPAQ) (0.45 to 0.65); and poor to fair criterion validity (0.06 to 0.35) [18].

### Sample size and sampling method

Based on the population census, there were 713,053 households or 1,666,888 people in Chiang Mai. The number of the targeted population, aged 15 to 64 years, was 1,247,376. A stratified two-stage cluster sampling method was used. First, the primary strata (Enumeration Areas) were classified identifying 24 urban and 12 rural areas. Second, 20 households in each area were randomly selected. Assuming a response rate of 80% and a design effect of 1.5, the calculated sample size to represent the targeted population in Chiang Mai was 1888.

### Variables and outcomes measurements

Demographic data on age and gender sex were collected. Age was categorised into 6 bands: 15–19; 20–29; 30–39; 40–49; 50–59 and 60–64. Highest education was used as a measure of socioeconomic status and was categorised into three categories: primary school, secondary school and college level.

Calculation of overall PA was done according to the guidelines on the Global Physical Activity Questionnaire (GPAQ) [12]. The participants were asked about the intensity, frequency and duration of the three domains of PA: 1) at work; 2) during travel or transport and 3) during recreational or leisure time. According to the GPAQ, a metabolic equivalent of task (MET) value of 4 was assigned for moderately intense PA and a value of 8 was assigned for vigorously intense PA. The assigned value MET was then multiplied by the number of days per week of PA and duration on a typical day for each domain of PA to create the amount of PA in metabolic equivalent of task-minutes per week (MET-minutes/week). The MET-minutes/week spent on each domain was then summed to create an overall PA level. According to the WHO guidelines, insufficient PA is defined as less than 600 MET-minutes/week of total energy expenditure from moderate or vigorous PA [12]. In addition, the GPAQ recommended collecting data on sedentary behaviour and sedentary time was collected in minutes per day.

For participants who answered that they did not participate in moderate and vigorous PA during their leisure time, the stage of change for LTPA was evaluated. To explore the related stages of change [19], an additional multiple choice question about plans and activities related to LTPA in the next 6 months was asked. Participants who chose “no current LTPA and no plan” were classified as in the ‘Pre-contemplation stage’. Those who chose “no current LTPA but planned to participate in LTPA in the next 6 months” were considered to be in the ‘Contemplation stage’ and those who chose “has some current LTPA but none at sufficient level of PA” were considered to be in the ‘Preparation stage’. The “action and maintenance stage” could not be applied to those who answered that they did not participate in moderate or vigorous LTPA.

### Statistical analysis

The demographic data of the participants was described using frequencies and percentages. The MET-minutes/week of energy used during PA among the study participants were presented as mean, standard deviation, medians, and interquartile range. To infer the estimates of the sample to the population, weighted analyses were performed to account for the aggregation of data. Stratified by age group and gender, the prevalence and 95% confidence interval (95% CI) of physical inactivity were estimated. The mean energy expenditure for overall PA and LTPA were estimated to consider the contribution of LTPA to overall PA. The difference of mean sedentary time between physically active and inactive populations was analysed using independent t-test. A subgroup analysis among those who were physical inactive was also performed to estimate the mean energy expenditure for overall PA and LTPA in this subgroup. The proportion of the population in each stage of change for LTPA among the physically inactive population were expressed as percentages and 95% CIs. Lastly, multinomial logistic regression was used to explore the influence of socioeconomic position on the stages of change among the physically inactive population. The statistical significance was considered when *p* < 0.05. All analyses accounted for the clustering of the data by using the survey command (*svy*) in STATA version 13 (StataCorp, Texas, USA).

## Results

### Study participants

A total of 1744 participants (response rate 92.4%) engaged in the study. Eight hundred and eight (46.3%) were men, and 936 (53.7%) were women. The mean age of the male participants was 43.6 and 43.3 for female participants. The sampled population represented the source population well (Table 1). The major group of the participants was from the sector of the population aged 50–59 (23.6%). The teenage group (aged 15–19) was a minority (5.3%) in this study.

**Table 1:** Proportion of men and women by age group in the sample and source population in Chiang Mai, Thailand, 2014 (row %)

	Men			Women		
Age group	Number of men in sample	Proportion of men in sample (row %)	Proportion of men in Chiang Mai (row %)	Number of women in sample	Proportion of women in sample (row %)	Proportion of women in Chiang Mai (row %)
15–19	35	37.6	41.1	58	62.4	58.9
20–29	128	48.9	50.8	134	51.1	49.2
30–39	138	50.2	49.9	137	49.8	50.1
40–49	162	41.6	40.9	227	58.3	59.1
50–59	235	46.0	46.3	276	54.0	53.7
60–64	110	51.4	52.1	104	48.6	47.9
All	808	46.3	46.8	936	53.7	53.2

### Prevalence of physical inactivity and sedentary time

**Fig. 1:**
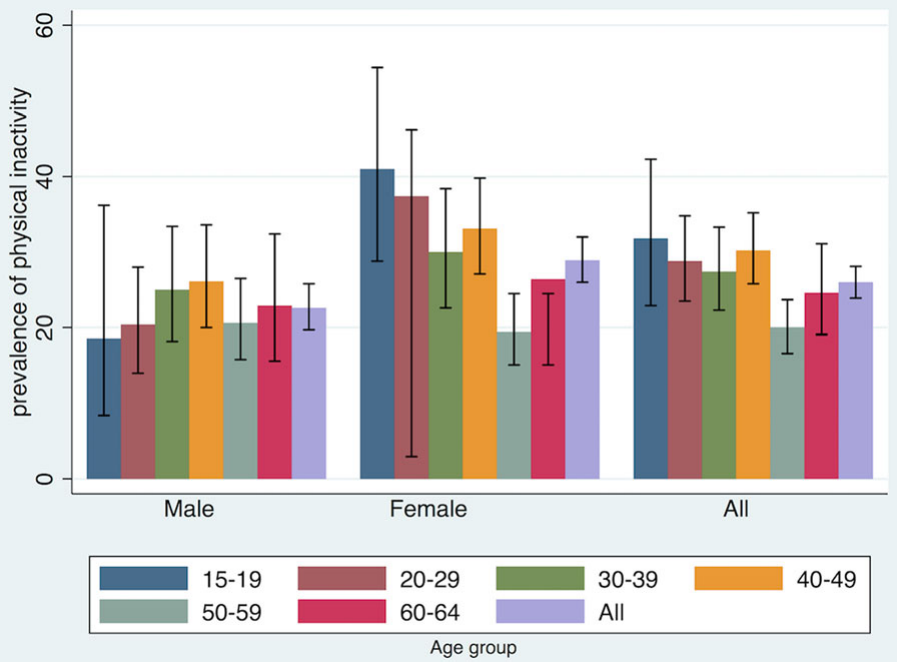
Prevalence and 95% confidence interval of physical inactivity by sex and age in Chiang Mai, Thailand, 2014

Overall, about 26.0% (95% CI 23.9 to 28.1) of the population was physically inactive. About 22.6% (95% CI 19.7 to 25.8) of men and 28.9% (95% CI 26.0 to 32.0) of women were physically inactive. Among men, the prevalence of physical inactivity was highest among those aged from 30 to 49, at approximately 25%. Among women, teenagers (aged 15–19) were the most inactive sector of the population (41.0%, 95% CI 28.8 to 54.4). Across most age groups, the prevalence of physical inactivity of women was higher than men (Fig. 1).

Overall, sedentary time did not statistically differ between the physically active and inactive population (Table 2). Among men, physically active population had an average of 134.0 min/day of time spent sitting. Inactive men had an average of 138.6 min/day of time spent sitting. Men aged over 60 had the highest sedentary time. For women, sedentary time was not statistically significant between active and inactive populations (124.6 min/week vs 134.0 min/week, *p* = 0.18). Teenage women spent the highest sedentary time in a day.

**Table 2:** Sedentary time among physically active and inactive populations in Chiang Mai, Thailand, 2014

Age group	Sedentary time (minutes/day) Mean (SD)					
	Men			Women		
	Active	Inactive	*p*-value	Active	Inactive	*p*-value
15–19	146.2 (106.1)	116.7 (95.0)	0.53	168.3 (94.4)	220.0 (112.8)	0.06
20–29	150.5 (78.9)	148.5 (121.3)	0.91	172.2 (108.4)	121.5 (112.6)	0.01
30–39	134.7 (81.2)	147.6 (152.5)	0.52	127.7 (87.1)	132.6 (109.0)	0.78
40–49	133.4 (81.4)	118.8 (110.1)	0.36	108.3 (75.5)	103.4 (104.6)	0.68
50–59	119.2 (79.5)	134.2 (113.7)	0.28	107.2 (79.4)	133.7 (110.4)	0.04
60–64	141.8 (93.3)	166.5 (133.7)	0.31	133.9 (91.1)	167.2 (118.7)	0.12
All	134.0 (83.9)	138.6 (123.5)	0.56	124.6 (88.7)	134.0 (113.8)	0.18

*SD* standard deviation

### Total energy use and energy use during leisure time

Table 3 demonstrates the total MET-minutes/week spent on overall PA and during LTPA. While total energy spent may be increasing with age when compared to the teenage population, the contribution of LTPA to overall PA level drastically decreased. Teenagers spent an average of 2538.9 MET-minutes/week on overall PA and 1397.8 MET-minutes/week on LTPA (55.0% of overall PA). In contrast, in those aged between 40 and 49, the average MET-minutes/week was at 7706.4 MET-minutes/week but LPTA only contributed 238.1 MET-minutes/week (3.1% of overall PA). Both men and women showed decreased contribution of LTPA to overall PA with advancing age (Table 3).

**Table 3:** Energy use on total physical activity and leisure time physical activity among study participants in Chiang Mai, Thailand, 2014 (*n* = 1744)

Age Group	Overall PA^a^			Leisure time PA		
	(MET-minutes/week)			(MET-minutes/week)		
	Men	Women	Total	Men	Women	Total
15–19						
Mean (SD)	3718.8 (4074.0)	1826.9 (3751.5)	2538.9 (3962.7)	2442.8 (2719.3)	767.2 (1391.1)	1397.8 (2144.3)
Median (IQR)	2690 (960 to 4860)	780 (120 to 1680)	1080 (240 to 2880)	1680 (360 to 3600)	280 (0 to 720)	480 (0 to 1920)
20–29						
Mean (SD)	8722.0 (9078.6)	3677.3 (5069.2)	6141.9 (7719.0)	1455.8 (2193.2)	488.3 (1218.1)	961.0 (1825.2)
Median (IQR)	5640 (1020 to 13,920)	1000 (180 to 7200)	2520 (420 to 10,080)	600 (0 to 2040)	0 (0 to 480)	0 (0 to 1120)
30–39						
Median (SD)	9253.0 (9198.5)	5549.8 (7932.5)	7480.1 (8773.8)	613.0 (1274.6)	193.1 (477.4)	403.8 (984.7)
Median (IQR)	6600 (720 to 15,120)	1500 (420 to 8400)	3360 (480 to 11,880)	0 (0 to 480)	0 (0 to 0)	0 (0 to 360)
40–49						
Mean (SD)	10,378.3 (10,100.1)	5799.6 (7314.1)	7706.4 (8865.6)	282.3 (734.0)	206.5 (625.2)	238.1 (672.7)
Median (IQR)	7540 (420 to 18,960)	1920 (400 to 10,080)	3840 (420 to 12,240)	0 (0 to 120)	0 (0 to 80)	0 (0 to 80)
50–59						
Mean (SD)	10,888.3 (10,765.5)	7546.4 (9.180.6)	9.083.3 (10,069.9)	232.9 (618.1)	274.8 (566.7)	255.5 (590.7)
Median (IQR)	7280 (960 to 20,160)	3110 (280 to 11,640)	4800 (840 to 14,000)	0 (0 to 0)	0 (0 to 330)	0 (0 to 240)
60–64						
Mean (SD)	7372 (9288.6)	4871.0 (6492.0)	6156.5 (8130.3)	319.6 (638.9)	271.9 (663.1)	296.4 (649.7)
Median (IQR)	2460 (860 to 10,860)	1860 (560 to 6720)	2260 (600 to 7960)	0 (0 to 360)	0 (0 to 240)	0 (0 to 280)
All						
Mean (SD)	9374.3 (9824.3)	5624.9 (7.657.0)	7362.0 (8923.9)	609.0 (1396.9)	307.0 (788.8)	446.9 (1122.5)
Median (IQR)	5640 (840 to 15,420)	1680 (480 to 8950)	3120 (560 to 11,760)	0 (0 to 480)	0 (0 to 240)	0 (0 to 360)

*SD* standard deviation, *IQR* inter-quartile range

^a^MET-min/week from overall PA is the sum of MET-min/week from the three domains of physical activity: 1) at work; 2) during travel or transport; and 3) during recreational activity or leisure time

### Physical activity and stages of change for leisure time physical activity in the physically inactive population

**Fig. 2:**
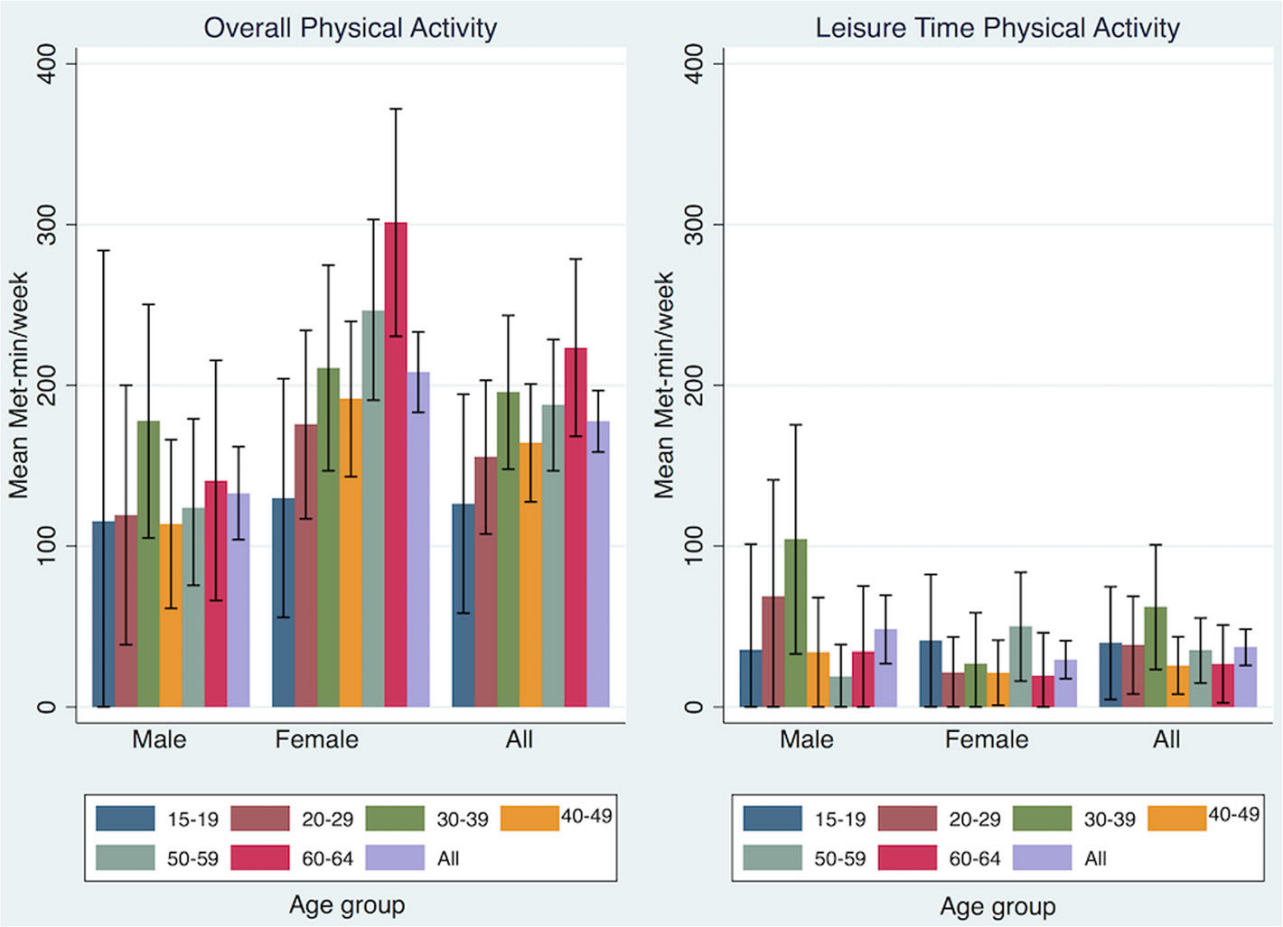
Mean MET-min/week expenditure for overall physical activity and for leisure time physical activity among the physically inactive population by age and sex in Chiang Mai, Thailand, 2014. MET-min/week from overall physical activity is the sum of MET-min/week from the three domains of physical activity: 1) at work; 2) during travel or transport; and 3) during recreational activity or leisure time

Among the physically inactive population in Chiang Mai, it is estimated that their average total energy expenditure was about only one-third of the recommended level of PA for women at 208.2 MET-minutes/week (95% CI 183.2 to 233.3) and only about one-fifth of the recommended level of PA for men at 132.8 MET-minutes/week (95% CI 104 .0 to 161.7). Similar to the findings from the overall population, LTPA only contributed to a small amount of overall PA. It is estimated that, on average, physically inactive men in Chiang Mai spent about 48.2 MET-minutes/week on LTPA (95% CI 27.0 to 69.4) while physically inactive women spent about 29.4 MET-minutes/week on LTPA (95% CI 17.7 to 41.1) (Fig. 2).

**Fig. 3:**
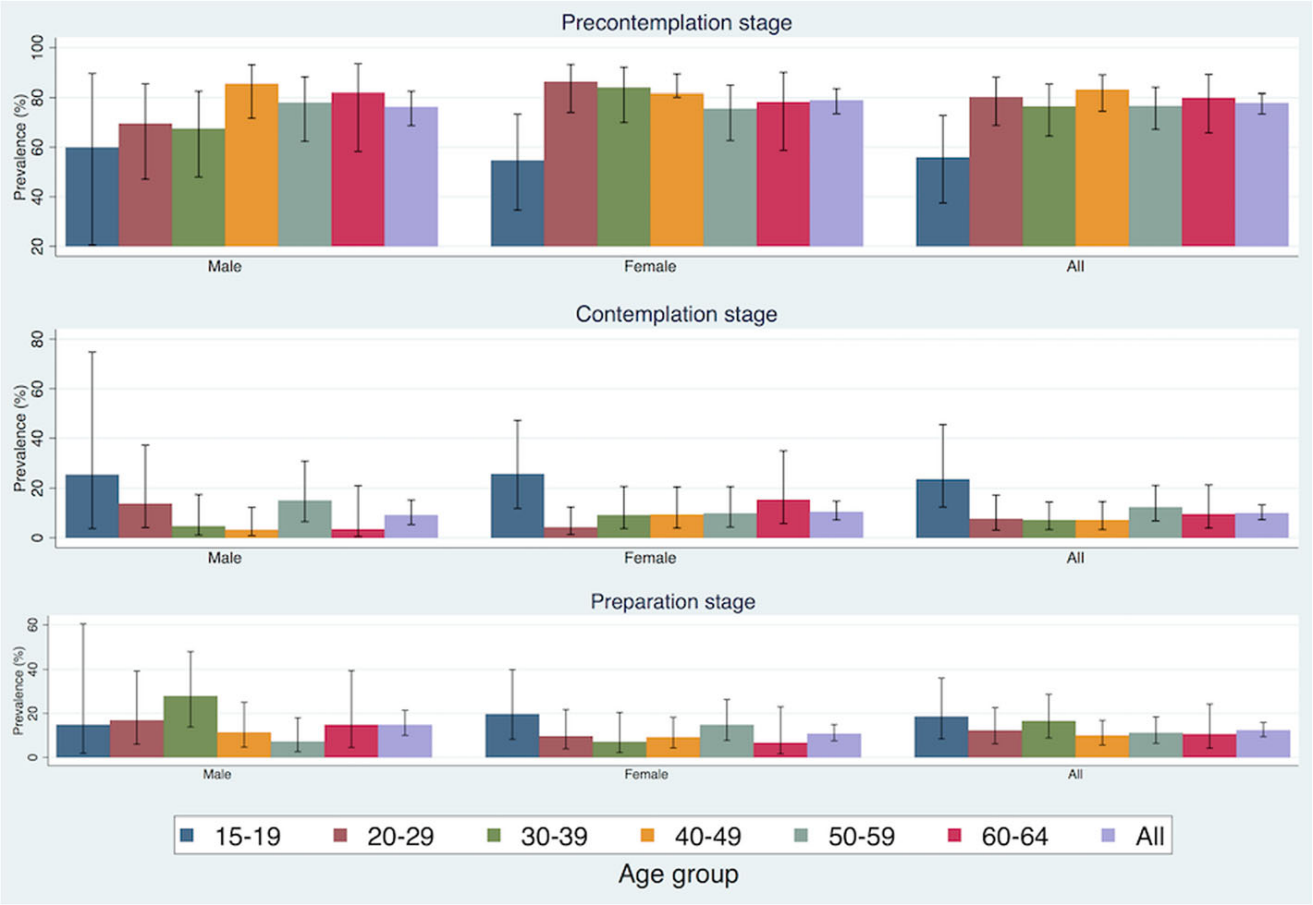
Stage of change for engaging in leisure time physical activity among physically inactive population by age and sex in Chiang Mai, Thailand, 2014

**Fig. 4:**
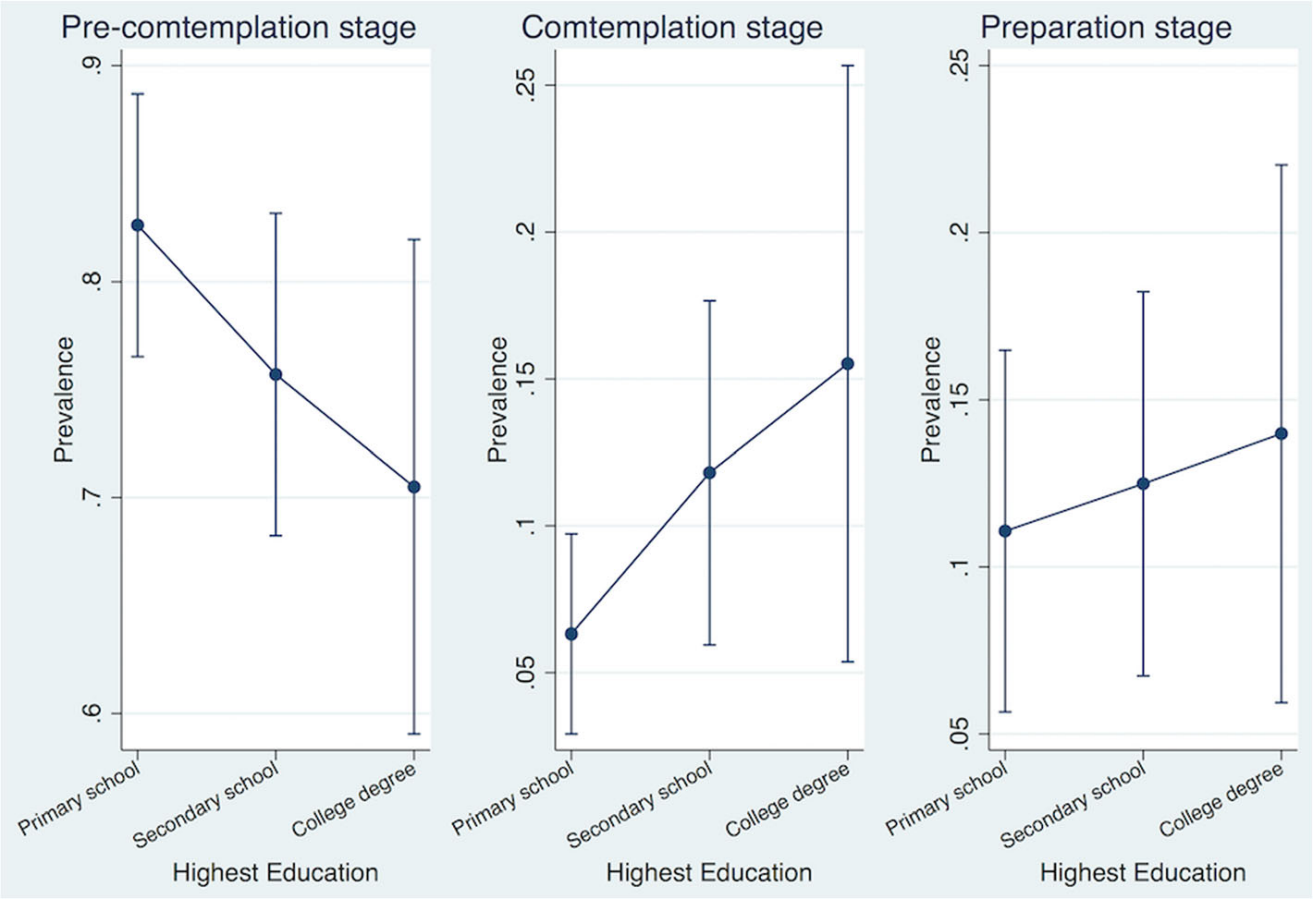
Highest education and stage of change for engaging in leisure time physical activity among physically inactive population in Chiang Mai, Thailand, 2014

Nearly 75% of the physically inactive population in Chiang Mai was in the pre-contemplation stage for engaging in PA during their leisure time. While about 14.7% of men (95% CI 10.0 to 21.4) and 10.7% of women (95% CI 7.5 to 14.9) were in the preparation stage (Fig. 3). While the results were not statistically significant, a trend was demonstrated that higher education could be inversely associated to being in pre-contemplation stage and positively associated with being in contemplation and preparation stage (Fig. 4).

## Discussion

The survey estimated that nearly a quarter of the population in Chiang Mai were physically inactive. This translates to more than 329,000 people aged from 15 to 64 failing to meet the recommended level on PA for health. It is estimated that, on average, over 400 MET-minutes/week are needed to get the physically inactive people in Chiang Mai over the 600 MET-minutes/week threshold set by WHO. As the contribution of LTPA to overall PA remained low for both the physically inactive population and even among the physically active population, targeting LTPA may be useful.

The prevalence of physical inactivity in Chiang Mai (26.0%) was similar to the overall level of physical inactivity in Thailand, estimated at 28.4% [8]. Similar to previous studies, our study found that women are less active than men [20–23]. It is possible that Thai women had more personal barriers, especially for outdoor activities, compared with men. A significant barrier to women’s outdoor PA was the sun light exposure due to cultural belief of the importance of fair skin [24].

The energy use of PA in a week of the population did not come primarily from LTPA and the contribution of LTPA to overall PA seemed to decrease with advancing age. This is supported by evidence from previous literature showing that young adults participate more in LTPA than do older age groups [25, 26]. The low level of LTPA among people living in Chiang Mai was similar to a study conducted in Vietnam. Trinh et al. [23] found that time spent in LTPA among Vietnamese people aged 25 to 64 years old was close to zero (6.7 min/day for men and 3.2 min/day for women).

The study explored the stages of change among the inactive population in Chiang Mai to understand the readiness and intention to change of this population. The first stage, the pre-contemplation stage, indicates no intention to change. The contemplation stage is a motivational stage without the actual behaviour and the preparation stage is a stage of strong motivation or tentative performance of the behaviour [13]. Our result was similar to another a population survey from the USA suggesting that socioeconomic indicators, such as education level, might be a determinant of intention to change. Lower educated population were more likely to be in pre-contemplation stage, whereas higher educated population had a greater probability of being in contemplation and preparation stages [14]. The understanding of the stages of change in the population can help to plan appropriate approaches for the population in each stage [27, 28]. Specifically, for those in the pre-contemplation stage, education and media campaigns can increase awareness toward the behaviour and stimulate positive emotions relating to the behaviour. For those in the contemplation stage, improvement of individuals’ cognition toward the behaviour can help progress towards the preparation stage. To step from the preparation stage to a later stage, individuals may need a strong commitment to change their behaviours [29, 30]. Woods et al. [31] reported that structural interventions, for example, sending the related material to individuals; encouraging individuals to consider the benefits of PA; asking individuals to commit to being active, can help inactive people to become more physically active. The study by Pirzadeh et al. [28] showed that educational sessions based on the stages of change including basic knowledge of PA, exercise training, and verbal encouragement can increase PA.

The global recommendation on PA [7] encourages people to increase overall PA, but it neither recommends a particular activity nor an intervention to promote PA in a large-scale population. For the whole population, a tailored intervention across all domains of PA might be a limitation because of the burden of resources. Studies by Williams et al. and Siegel et al. demonstrated that walking can be a popular, acceptable, and accessible way to promote LTPA [32, 33]. Mass media, such as television advertisements; printed media; telephone or internet-based interventions, can increase awareness of LTPA participation [32, 34]. Group walking is also an efficacious approach of increasing PA [35].

Furthermore, evidence showed that in Belgium, a multi-strategy community-based intervention including local media campaign, environmental approaches, the sale and loan of pedometers, and several local PA projects was effective to increase PA levels [36]. Built environments are also a key aspect to increase PA in a large-scale population. More and better-quality of sidewalks and bicycle lanes are associated with higher rates of walking, biking and meeting PA recommendations [37]. Active transport or non-motorised transport is associated with increases of PA and positive health outcomes [38]. Moreover, natural environments such as parks, woodlands and beaches are keys locations for PA [39]. Accordingly, there are many effective approaches to increase PA during work, travel and leisure time, however, targeting LTPA may be useful in the promotion of PA among the population as there may be fewer un-modifiable barriers than PA at work and during transport.

The strengths of this study were the systematic method of sampling, data collection, and coverage of study participants. The study was able to represent different populations in various age groups from both urban and rural areas. There were some limitations of this study. Although the survey used a reliable and valid questionnaire for PA, GPAQ [18, 40], collection of the PA data by a self-reporting questionnaire was subjective [41] and may depend on the recall memory of the participant [42]. This cross-sectional study could not assess the outcomes of intentions to change among people who were physically inactive. Lastly, detailed socioeconomic status and the health related data, for instance, body weight; body mass index; and blood pressure was not collected thus we were not able to correlate PA levels with health outcomes.

According to our findings, the contribution of LTPA to overall PA among physically inactive population remained consistently low across all age groups, and approximately 400 MET-minutes/week of PA or at least 100 min/week of moderate-intensity LTPA or at least 50 min/week of vigorous-intensity LTPA should be promoted to the physically inactive population in Chiang Mai. We recommend that any PA promotion programmes need multistrategy approaches including supportive environments, media campaigns and psychosocial supports population in the pre-contemplation stage, along with increasing education and awareness of the importance of PA through media campaigns.

Follow up data on the intention to change and PA level is recommended to monitor trends and changes in the PA level of the population. Other data on personal and environmental circumstances may also be useful to effectively plan for interventions to promote PA in a large population. As the impact of PA on health outcomes may vary by settings we recommend collecting basic health measurements, for example, blood pressure; body weight and height and BMI which may be beneficial in assessing the impact of changes in PA on health related outcomes.

## Conclusions

It is estimated that about a quarter of the Chiang Mai population, or approximately 329,000 people living in Chiang Mai, were physically inactive during 2014. The contribution of LTPA to overall PA was low among the physically inactive population, and the majority has no intention of increasing LTPA. A large-scale health promotion programme is needed, and it should focus on an approach for the sector of the population in the pre-contemplation stage.

## Abbreviations

BMI: Body mass index
CI: Confidence interval
GPAQ: Global Physical Activity Questionnaire
LTPA: Leisure time physical activity
MET: Metabolic equivalent of task
NCD: Non-communicable disease
PA: Physical activity
WHO: World Health Organization
